# National estimates of mortality of unintentional drowning in China from 1990 to 2021 and its predicted level in the next decade: results from the global burden of disease study 2021

**DOI:** 10.3389/fpubh.2025.1533173

**Published:** 2025-03-19

**Authors:** Liuqing You, Jiangmei Liu, Jieming Zhong, Fangrong Fei

**Affiliations:** ^1^Zhejiang Center for Disease Control and Prevention (Zhejiang CDC), Hangzhou, China; ^2^National Center for Chronic and Noncommunicable Disease Control and Prevention, Chinese Center for Disease Control and Prevention, Beijing, China

**Keywords:** unintentional drowning, global disease burden, deaths, decomposition, prediction

## Abstract

**Background:**

It is reported that burden of unintentional drowning deaths is high in low- and middle-income countries. In recent decades, China has achieved remarkable economic growth and substantial advancements in infrastructure development; however, the understanding of the unintentional drowning burden in China has lagged behind. This article aims to provide an in-depth understanding of the current unintentional drowning situation in China.

**Methods:**

Unintentional drowning from GBD 2021 was estimated for cause-specific mortality and, age, sex, and temporal trends from 1990 to 2021. In addition, we used decomposition analysis to quantify the drivers of changes in unintentional drowning from 1990 to 2021 and we also predicted the mortality of unintentional drowning in the next 10 years based on APC model.

**Results:**

In 2021, the deaths attributable to unintentional drowning in China were 57554.02 (95% UI: 47463.15~69111.96), corresponding to age-standardized mortality rate (ASMR) of 4.12 (95% UI: 3.39 ~ 4.96) per 100,000 population. The mortality rate was relatively high among children aged 0–10 years and individuals aged 60 years and above and the highest number of deaths were recorded in the age groups of <5 years (3753.78, 95% UI: 2834.88 ~ 4903.46), 5–9 years (4938.93, 95% UI: 4207.74 ~ 5751.58), and 10–14 years (4197.10, 95% UI: 3581.12 ~ 4819.72). The mortality of unintentional drowning was higher for males than females across all age groups. A decline in unintentional drowning mortality rates was observed from 1990 to 2021, with an average annual percentage change (AAPC) of −4.19%. Epidemiological changes were the primary contributors to the observed decline in unintentional drowning deaths (decreased by 124985.81). The ASMR of unintentional drowning would continue to decrease slowly at the national level and that the decreasing trends would be stable in the future.

**Conclusion:**

From 1990 to 2021, the mortality rate of unintentional drowning in China showed a downward trend. Males, children under 10 years old, and older adult people aged 65 and above were identified as high-risk factors for drowning. The research findings emphasize the importance of continuing to strengthen data collection systems, identifying risk factors, and developing drowning prevention strategies tailored to China’s national conditions.

## 1 Introduction

Drowning, the process of experiencing respiratory impairment from submersion/immersion in liquid (1), poses a significant challenge to global public health (2). It has been reported that, unintentional drowning has become the third leading cause of unintentional injury deaths worldwide, accounting for approximately 9% of all injury-related deaths (3, 4). According to a report by the World Health Organization (WHO), it is estimated that about 300,000 people died from drowning every year (5), and behind this number are countless family tragedies and a heavy social burden.

From both economic and geographic perspectives, the issue of unintentional drowning is particularly severe in low- and middle-income countries, where the number of unintentional drowning deaths accounts for more than 90% of the global burden (6). Additionally, over half of the global drowning incidents occur in the WHO Western Pacific Region and the WHO Southeast Asia Region (7, 8). This geographical disparity reflects the close correlation between unintentional drowning and factors such as economic development level, water resource management, and public safety awareness (9, 10).

China, as the largest developing country, has a large and widely distributed population with a relatively high population density (11). It also boasts diverse topography and abundant water resources. These characteristics provide potential environmental and demographic conditions for the occurrence of drowning incidents in China. It has been reported that, drowning was the fourth leading cause of injury-related deaths in China in 2017, with a prominent issue of childhood drowning (12). In recent decades, China has undergone remarkable economic growth and substantial advancements in infrastructure development and also tried to develop a medium-term health strategy that includes drowning as an injury (13). Despite these significant progressions, the current understanding of the unintentional drowning situation in China has lagged behind.

This research presents a comprehensive overview to further understand the magnitude of unintentional drowning as a public health problem in China. Specifically, this study aims to (i) examine the mortality of fatal unintentional drowning across different age groups and sexes, (ii) explore their temporal trends and the drivers of change, and (iii) predict the mortality rates for the next 10 years in China, using the 2021 Global Burden of Disease (GBD) Study estimates.

## 2 Methods

### 2.1 Data source

The GBD is the largest and most comprehensive study to measure the epidemiological levels and trends worldwide (14). The GBD study used a Bayesian meta regression tool (DisMod-MR V.2.1), to pool the heterogeneous data. Detailed methodology for the GBD Study 2021 is available in other sources (15, 16). This current study focuses on unintentional drowning deaths, and the GBD Study 2021 used the ICD-9 code, E910 and ICD-10 codes (W65–W74) for unintentional drowning (4, 17). We extract unintentional drowning deaths in China: mortality rates, based on year, sex, age group (all ages, and by 5-year age groups).

To gain a more nuanced understanding of China’s unintentional drowning situation within the global context and to compare it with other countries and regions, countries and territories were also categorized into quintiles of high, high-middle, middle, low-middle, and low Socio-demographic index (SDI). The SDI is a composite indicator with scores from 0 to 1, based on the geometric mean of a region’s lag-distributed income per capita, the average years of schooling for the population aged 15 and above, and the total fertility rate of women under the age of 25 years (18), on the basis of their 2021 values. This study was approved by the ethics committee of the Zhejiang Provincial Center for Disease Control and Prevention.

### 2.2 Statistical analysis

Age-standardized mortality rates (ASMR) and their uncertainty interval (UI) were presented using the GBD world population age standard as a reference (19). Direct standardization yields age-adjusted rates, which are weighted averages of age-specific rates, representing the relative age distribution. The ASMR = *Σ* (age-specific mortality rate × the composition ratio of that age group in the standard population). Estimated annual percentage change (EAPC) and its 95% confidence interval (CI) were calculated.

A Joinpoint regression analysis was conducted to track the changes in mortality of unintentional drowning over time and pinpoint years with significant shifts in the trend and this method allowed us to calculate the average annual percentage change (AAPC) across different trend segments with the optimal number of segments determined by a permutation test (20).

We used decomposition analysis to quantify dynamics of unintentional drowning deaths burden over the study period (21). This method dissects the whole variation into three components: epidemiological changes (i.e., changes due to changes in GBD risk factor prevalence, like environmental factors and behavior factors), population growth, and aging (i.e., population age structure) (22, 23).

Based on the age-period-cohort (APC) model, we then predict mortality rates of unintentional drowning from 2021 to 2031, using Nordpred R package (24). To facilitate a comparison with predicted outcomes, a Bayesian age-period-cohort (BAPC) model using an integrated nested Laplace approximation (INLA) was performed to verify the stability of the predicted results. The analysis was conducted using R software, version 4.0.1 and all testing was two-sided with statistical significance set at *α* = 0.05.

## 3 Results

The deaths number attributable to unintentional drowning in China was 57554.02 (95% UI: 47463.15 ~ 69111.96) in 2021, corresponding to ASMR of 4.12 (95% UI: 3.39 ~ 4.96) per 100,000 population (Table 1). The estimated EAPC from 1990 to 2021 was −3.86% (95% CI: −4.05% ~ −3.67%) (Table 1). The global ASMR for unintentional drowning is 3.56 (95% UI: 3.12 ~ 4.01) per 100,000 population, and it shows a decreasing trend with an increase in SDI (Table 1).

**Table 1:** Absolute deaths number and age-standardized mortality rates per 100,000 population from unintentional drowning in 2021, and estimated annual percentage changes (EAPC) for 1990–2021, by SDI Level.

Regions	Absolute deaths number in 2021 (95% UI)	Age-standardized mortality rates (95% UI)	EAPC (95% CI)
China	57554.02 (47463.15, 69111.96)	4.12 (3.39, 4.96)	−3.86 (−4.05, −3.67)
Global	274230.18 (242192.92, 307179.57)	3.56 (3.12, 4.01)	−2.79 (−2.89, −2.70)
Low SDI	57443.13 (44994.74, 70171.28)	5.25 (4.26, 6.26)	−2.00 (−2.05, −1.94)
Low-middle SDI	77707.92 (66939.26, 89341.57)	4.25 (3.66, 4.88)	−2.56 (−2.67, −2.46)
Middle SDI	83109.33 (73471.73, 94045.42)	3.52 (3.10, 4.00)	−3.43 (−3.50, −3.37)
High-middle SDI	35586.97 (31103.12, 40564.11)	2.56 (2.24, 2.93)	−3.45 (−3.76, −3.14)
High SDI	20153.18 (17768.08, 21866.72)	1.31 (1.18, 1.42)	−2.12 (−2.18, −2.05)

**Figure 1:**
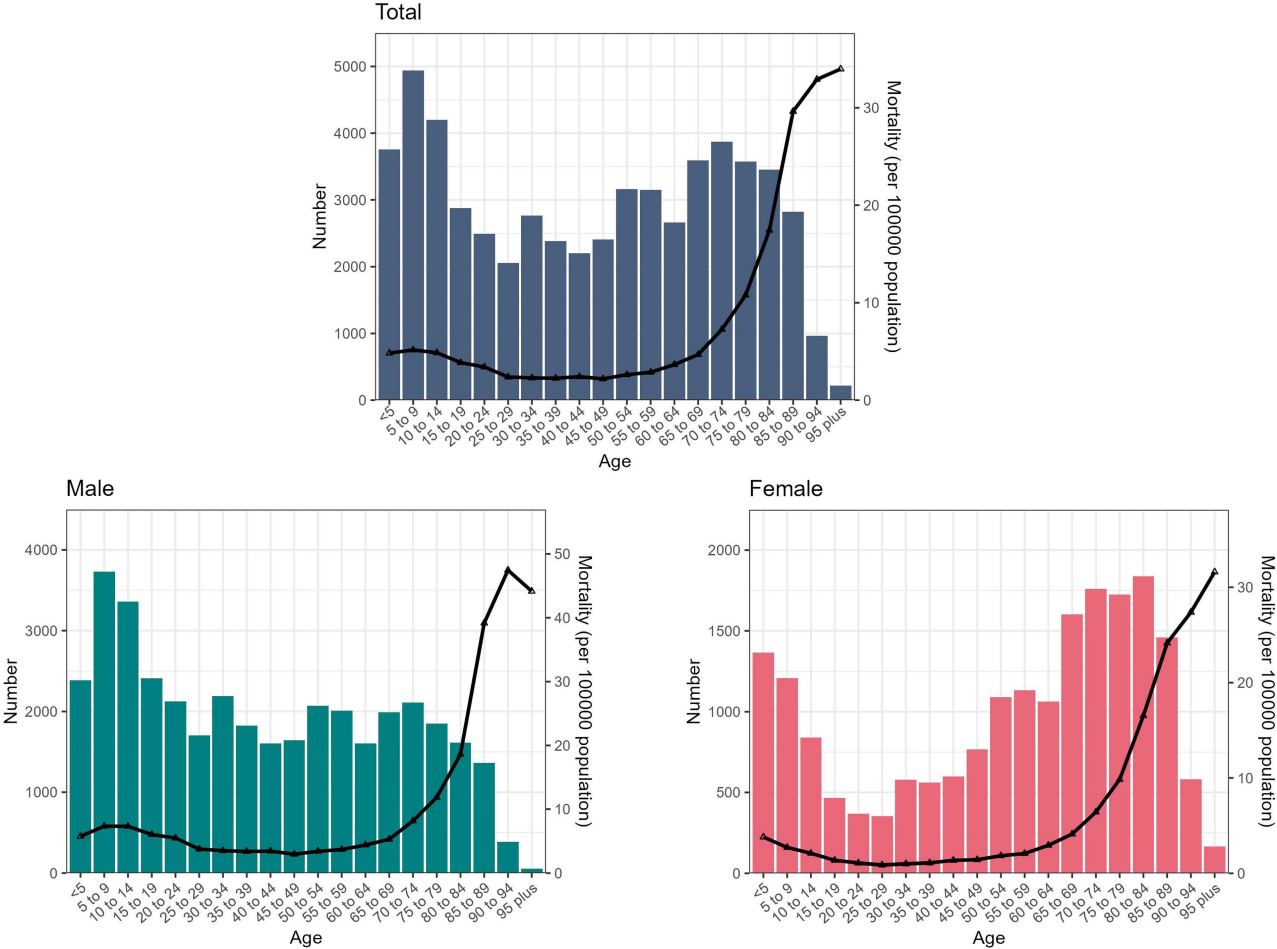
The absolute number and age-specific mortality in China in 2021 for both sexes and all ages.

In 2021, the mortality was relatively high among children aged 0–10 years (≥4.83 (95%UI: 3.65 ~ 6.31) per 100,000 population) and individuals aged 60 years and above (≥3.65 (95% UI: 2.99 ~ 4.42) per 100,000 population) (Figure 1). The mortality of unintentional drowning was higher for males than females across all age groups (Figure 1). In terms of absolute numbers, the highest number of deaths were recorded in the age groups of <5 years (3753.78, 95% UI: 2834.88 ~ 4903.46), 5–9 years (4938.93, 95% UI: 4207.74 ~ 5751.58), and 10–14 years (4197.10, 95% UI: 3581.12 ~ 4819.72) (Figure 1). More detailed numerical values were provided in Supplementary Table S1.

**Figure 2:**
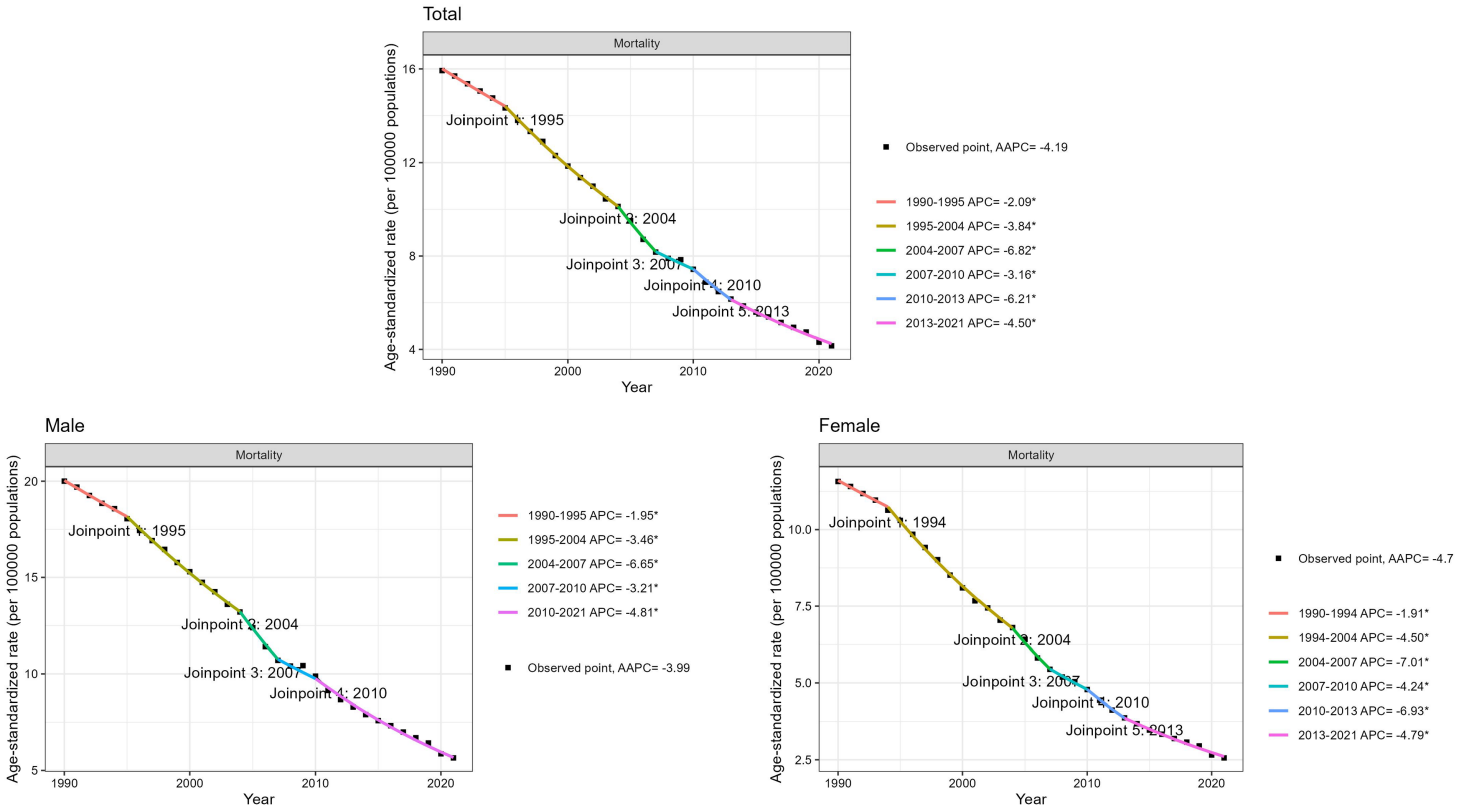
Joinpoint regression analysis of the age-standardized mortality rate (ASMR) for unintentional drowning in China from 1990 to 2021. The black dots represent the ASMR for the corresponding years, while the endpoints of different colored segments mark the turning points in the trend.

During the period from 1990 to 2021, a decline in total unintentional drowning mortality rate was observed, with an AAPC of −4.19% (Figure 2). Both males and females exhibited a downward trend, with AAPCs of −3.99% and −4.70%, respectively (Figure 2). More detailed numerical values were provided in Supplementary Table S2.

**Figure 3:**
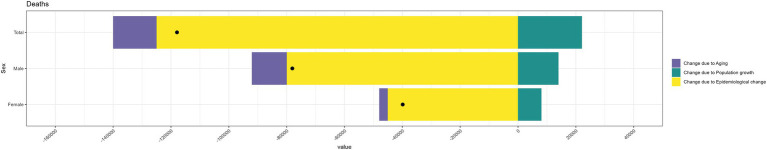
Decomposition analysis of the unintentional drowning deaths in China from 1990 to 2021. The black dot represents the overall difference, that is, the reduction in unintentional drowning deaths in 2021 compared to 1990. The bars of different colors indicate the contributions of three factors to the number of unintentional drowning deaths: changes due to aging, population growth, and epidemiological change. The sum of the changes contributed by these three factors equals the overall difference value represented by the black dot.

In 2021, unintentional drowning deaths decreased by 117916.70 compared to 1990, with epidemiological changes being the primary factor contributing to this reduction (decreased by 124985.81). The population aging also played a negative impact (decreased by 15023.77) on the unintentional drowning death burden and population growth exerted a positive impact (increased by 22092.88) on the unintentional drowning deaths burden. For males, in comparison to 1990, the overall difference in unintentional drowning deaths in 2021 was −78040.35. Specifically, the changes attributed to epidemiological changes, population aging, and population growth were −79948.20, −12072.23, and 13980.08, respectively (Figure 3).

**Figure 4:**
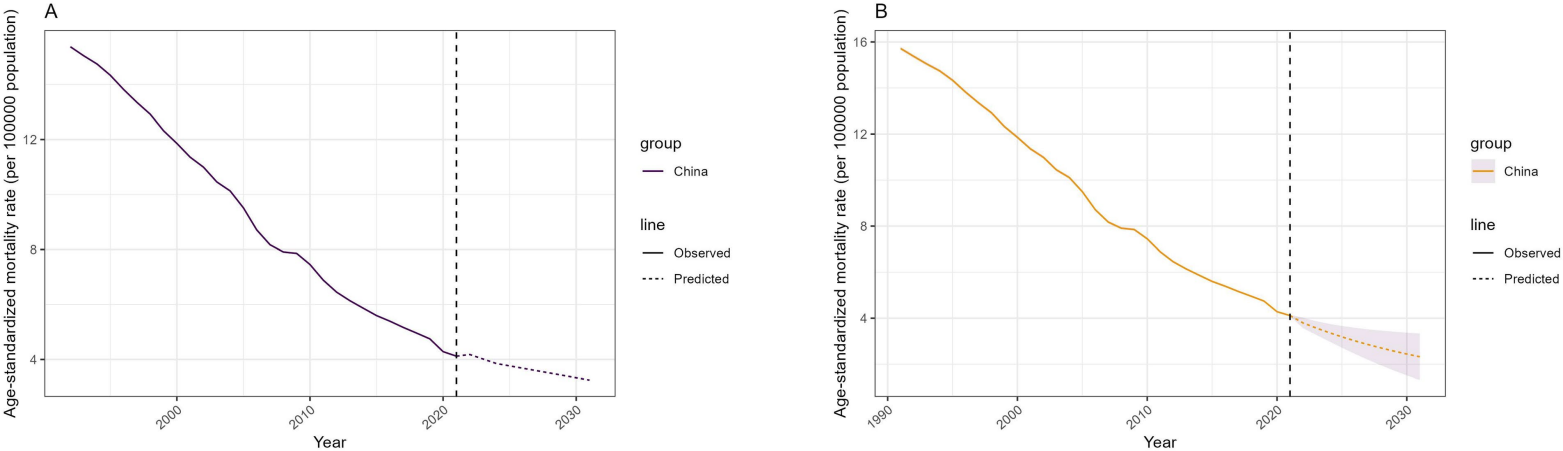
Predicted trends in the age-standardized unintentional drowning in the next decades in China. **(A)** Nordpred model. **(B)** Bayesian age-period-cohort model.

It is expected that the global ASMR of unintentional drowning will slowly decrease from 2021 to 2031. The ASMR of total unintentional drowning will decline slowly, from 4.12 per 100,000 population in 2021 to 3.33 per 100,000 population in 2031. The prediction results of the BAPC model exhibited a similar trend (Figure 4).

## 4 Discussion

Our analysis of data from the most recent GBD study revealed that in 2021, the deaths attributable to unintentional drowning in China were 57554.02. Corresponding ASMR were 4.12%. These figures were comparable to those observed in low-middle SDI regions and exceed global averages, aligning with previous research (25). Despite China’s significant progress in economic and social development, unintentional drowning remains a public health issue.

From a sex perspective, between 1990 and 2021, the mortality rate among Chinese males was higher than that of females in most age groups, which was consistent with research findings observed in other regions previously (26, 27). This might be related to the higher self-efficacy of males, who are more willing to take risks and attempt adventurous behaviors (28). Therefore, further research on the contributing factors and protective factors related to the risk of drowning is crucial. In terms of age distribution, we found that the mortality of unintentional drowning in children under 10 and adults over 65 years exceed 4.5 per 100,000 population. The high mortality of unintentional drowning in children under 10 years was consistent with the internationally recognized awareness of the high-risk age groups for drowning death (29, 30). This might be attributed to their curiosity and desire for water but lack of awareness of its dangers and poor parental supervision (2, 31). Compared to unintentional drowning death in children, limited attention had been paid to unintentional drowning deaths in the older adult. The high unintentional drowning mortality among the older adult may be related to their declining physical capabilities (32). Previous studies indicated that as individuals age, they experience a reduction in muscle mass and strength, coupled with a decline in physical flexibility (33, 34). These physiological changes can impair the ability of older adults to effectively respond to emergency situations, including drowning (35). However, recent paper revealed a lack of consensus regarding the risk factors for unintentional drowning among older adults (36). The findings of the current study should be a call to action to invest in drowning prevention among older people in China.

The joinpoint regression results indicated that the ASMR for unintentional drowning, showed an overall decreasing trend over the past three decades. The decomposition analysis indicated that the epidemiological changes of unintentional drowning was the primary contributors to the observed decline in deaths. The rapid urbanization process in China over the past few decades might be a significant factor contributing to the decline in unintentional drowning mortality (37, 38). Urbanization trend has limited public interaction with natural water sources, which were identified as major risk factors for unintentional drowning, with most drownings in China occurring in such waters (39, 40). Urbanization also had shifted people’s activities and entertainment from outdoor to safer indoor environments (41). Simultaneously, urbanization also brings changes in education, such as the increased accessibility to safety education, which may play a significant role in preventing drowning (42). Studies have demonstrated that individuals with advanced education are more likely to implement comprehensive safety practices and opt for safer venues for aquatic activities (43, 44). The results of the current study indicated that certain achievements have indeed been made in the current efforts to prevent unintentional drowning deaths. Therefore, it was necessary to explore the impact of socioeconomic progress and evaluate which intervention measures can effectively reduce the burden of unintentional drowning deaths in China.

Our study conducted predictions on unintentional drowning mortality in China in the next decade. The results revealed a concerning trend: China’s drowning mortality rate might remain higher than the global average even 10 years into the future, and the rate of decline appears to be slowing down over time. This projection underscores the urgent need for novel and innovative measures to effectively address the persisting issue of unintentional drowning in China. Currently, the death cause monitoring system in China employs the International Classification of Diseases (ICD) system, specifically ICD-10, to categorize and report causes of death (45). Although its widespread use and standardization, it still has certain limitations in the classification and coding of drowning. Specifically, the current ICD-10 coding system (codes W65-W74) primarily focuses on the location of drowning (e.g., swimming pools, bathtubs, natural water bodies, etc.), but fails to adequately reflect the specific contextual characteristics and causal mechanisms of drowning events. For instance, in cases of infant drowning, incidents involving common household containers such as buckets or water tanks are often broadly categorized, making it impossible to capture their unique environmental features and prevention priorities. Therefore, establishing a more detailed investigation and coding system for drowning incidents, particularly by incorporating key elements such as the type of container, water depth, and supervision status, will help more accurately identify risk factors and develop targeted prevention strategies, thereby enhancing the scientific rigor and effectiveness of unintentional drowning prevention efforts. It is also necessary to explore and integrate additional data sources and methodologies that can provide a more granular and holistic view of drowning incidents in China (46, 47).

## 5 Strengths and limitations

This study holds notable importance in exploring the issue of drowning in China, particularly considering that it has been nearly a decade since the last update in this field, which was the GBD 2013 study.

Despite the progress made in addressing the issue of unintentional drowning deaths, our study still faces certain limitations. Firstly, the data of the GBD study mainly depend on the reporting and statistical systems of various countries and regions, which may vary in accuracy and completeness (48). Moreover, the ICD-10 coding used by the GBD study may not fully capture the complexity and details of drowning incidents, such as environmental factors involved in drowning events. Therefore, the research results may be limited by the data sources and analytical methods, and may not fully reflect the true situation of drowning deaths. Secondly, the lack of specific data from sectors such as education and construction, as well as from the provincial level, and detailed information on drowning location, hinders our in-depth analysis of different regions, populations, educational backgrounds, urbanization levels, and drowning sites.

While this study has provided insight into the issue of unintentional drowning deaths in China, several key areas for future research are noted: (1) Enhancing the drowning mortality surveillance system to better capture the intricacies and detailed aspects of unintentional drowning incidents with greater precision. (2) Integrating data across various departments to elevate the quality and depth of research endeavors, while exploring innovative methods to advance the prevention and control of unintentional drowning. (3) Highlighting the significance of unintentional drowning deaths among the older adult population and initiating an investigation into these incidents, along with their corresponding risk factors.

## 6 Conclusion

From 1990 to 2021, the mortality rate of unintentional drowning in China showed a downward trend. Males, children under 10 years old, and older adult people aged 65 and above were identified as high-risk factors for drowning. The research findings emphasize the importance of continuing to strengthen data collection systems, identifying risk factors, and developing drowning prevention strategies tailored to China’s national conditions.

## Data Availability

Publicly available datasets were analyzed in this study. This data can be found at: The GBD 2021 repository, which is freely available at http://ghdx.healthdata.org/gbd-results-tool.
