# Supplementary material for: National estimates of mortality of unintentional drowning in China from 1990 to 2021 and its predicted level in the next decade: results from the global burden of disease study 2021
Source: Front Public Health. 2025 Mar 19;13:1533173. doi: 10.3389/fpubh.2025.1533173 (PMC11961984; doi:10.3389/fpubh.2025.1533173)
Supplement: Supplementary file 1 [file Table_1.docx]

| Supplement Table S1 The Mortality of Unintentional Drowning in China in 2021 | | | | | | |
| --- | --- | --- | --- | --- | --- | --- |
|  | **Both** | | **Male** | | **Female** | |
| **Age** | **Number** | **Rate (per 100000)** | **Number** | **Rate (per 100000)** | **Number** | **Rate (per 100000)** |
| <5 | 3753.78 (2834.88, 4903.46) | 4.83 (3.65, 6.31) | 2387.39 (1798.71, 3123.18) | 5.73 (4.32, 7.50) | 1366.39 (1014.37, 1763.35) | 3.79 (2.81, 4.89) |
| 5 to 9 | 4938.93 (4207.74, 5751.58) | 5.16 (4.39, 6.01) | 3729.64 (3160.64, 4347.93) | 7.32 (6.20, 8.53) | 1209.30 (1021.18, 1422.74) | 2.70 (2.28, 3.17) |
| 10 to 14 | 4197.10 (3581.12, 4819.72) | 4.87 (4.15, 5.59) | 3357.84 (2793.14, 3939.88) | 7.30 (6.07, 8.57) | 839.26 (703.79, 1006.15) | 2.09 (1.75, 2.50) |
| 15 to 19 | 2878.60 (2364.10, 3501.65) | 3.85 (3.17, 4.69) | 2412.65 (1915.13, 3015.14) | 6.02 (4.78, 7.52) | 465.95 (390.12, 557.57) | 1.35 (1.13, 1.61) |
| 20 to 24 | 2494.78 (2046.80, 3058.31) | 3.41 (2.80, 4.18) | 2126.36 (1688.63, 2675.54) | 5.47 (4.34, 6.88) | 368.41 (278.37, 472.24) | 1.07 (0.81, 1.38) |
| 25 to 29 | 2055.02 (1746.31, 2396.96) | 2.38 (2.02, 2.77) | 1703.28 (1423.59, 2032.02) | 3.73 (3.12, 4.45) | 351.74 (259.59, 468.12) | 0.86 (0.64, 1.15) |
| 30 to 34 | 2767.06 (2358.31, 3262.46) | 2.28 (1.95, 2.69) | 2189.29 (1793.44, 2658.27) | 3.49 (2.86, 4.24) | 577.77 (437.32, 758.14) | 0.99 (0.75, 1.30) |
| 35 to 39 | 2386.65 (1985.66, 2863.57) | 2.25 (1.87, 2.70) | 1826.37 (1453.09, 2260.00) | 3.36 (2.67, 4.16) | 560.28 (415.97, 736.15) | 1.09 (0.81, 1.43) |
| 40 to 44 | 2203.02 (1801.54, 2709.09) | 2.41 (1.97, 2.96) | 1603.21 (1229.64, 2050.14) | 3.42 (2.62, 4.37) | 599.82 (448.73, 779.78) | 1.34 (1.01, 1.75) |
| 45 to 49 | 2411.07 (1960.73, 2991.86) | 2.19 (1.78, 2.71) | 1643.94 (1233.64, 2183.50) | 2.93 (2.20, 3.89) | 767.14 (571.23, 1008.15) | 1.41 (1.05, 1.86) |
| 50 to 54 | 3162.11 (2558.55, 3880.25) | 2.62 (2.12, 3.21) | 2072.54 (1528.40, 2783.25) | 3.39 (2.50, 4.55) | 1089.57 (826.48, 1395.94) | 1.82 (1.38, 2.34) |
| 55 to 59 | 3146.84 (2542.62, 3859.25) | 2.86 (2.31, 3.51) | 2012.68 (1503.51, 2662.71) | 3.67 (2.74, 4.85) | 1134.17 (864.10, 1483.91) | 2.06 (1.57, 2.70) |
| 60 to 64 | 2665.73 (2182.50, 3228.81) | 3.65 (2.99, 4.42) | 1603.11 (1198.44, 2083.80) | 4.38 (3.27, 5.69) | 1062.62 (835.11, 1340.65) | 2.92 (2.30, 3.69) |
| 65 to 69 | 3592.48 (2989.47, 4304.30) | 4.68 (3.90, 5.61) | 1990.64 (1513.19, 2584.19) | 5.27 (4.01, 6.85) | 1601.84 (1261.63, 2005.35) | 4.11 (3.24, 5.15) |
| 70 to 74 | 3871.21 (3189.00, 4580.12) | 7.26 (5.98, 8.59) | 2110.04 (1630.87, 2681.01) | 8.16 (6.31, 10.37) | 1761.17 (1383.40, 2202.69) | 6.42 (5.04, 8.03) |
| 75 to 79 | 3571.72 (2971.96, 4236.08) | 10.78 (8.97, 12.79) | 1847.91 (1464.53, 2378.32) | 11.84 (9.39, 15.24) | 1723.81 (1334.44, 2175.43) | 9.84 (7.62, 12.42) |
| 80 to 84 | 3452.80 (2869.88, 4075.64) | 17.45 (14.5, 20.59) | 1613.95 (1289.28, 1981.09) | 18.60 (14.86, 22.84) | 1838.85 (1444.28, 2263.05) | 16.54 (12.99, 20.36) |
| 85 to 89 | 2822.85 (2333.87, 3297.97) | 29.63 (24.5, 34.62) | 1363.31 (1174.84, 1610.42) | 39.17 (33.75, 46.26) | 1459.54 (1084.64, 1848.59) | 24.15 (17.94, 30.58) |
| 90 to 94 | 965.07 (772.45, 1127.58) | 32.92 (26.35, 38.46) | 383.83 (316.20, 456.78) | 47.45 (39.09, 56.47) | 581.24 (426.78, 722.20) | 27.38 (20.10, 34.02) |
| 95 plus | 217.18 (165.65, 263.28) | 33.98 (25.92, 41.19) | 53.40 (44.05, 61.47) | 44.15 (36.42, 50.82) | 163.78 (119.33, 207.27) | 31.61 (23.03, 40.00) |

| **Supplement Table S2 The Age-standardized mortality of Unintentional Drowning in China from 1990 to 2021** | | | |
| --- | --- | --- | --- |
|  | **Age-standardized mortality (per 100 000)** | | |
|  | **Both** | **Male** | **Female** |
| **Year** | **Rate (per 100000)** | **Rate (per 100000)** | **Rate (per 100000)** |
| 1990 | 15.93 (14.11, 18.13) | 20.00 (17.65, 22.83) | 11.57 (10.15, 13.16) |
| 1991 | 15.70 (14.09, 17.49) | 19.69 (17.47, 21.97) | 11.41 (10.17, 12.94) |
| 1992 | 15.36 (13.84, 17.08) | 19.26 (17.17, 21.39) | 11.18 (9.93, 12.68) |
| 1993 | 15.05 (13.62, 16.71) | 18.85 (16.98, 20.91) | 10.96 (9.75, 12.35) |
| 1994 | 14.75 (13.34, 16.27) | 18.57 (16.72, 20.46) | 10.64 (9.41, 11.92) |
| 1995 | 14.33 (13.12, 15.65) | 18.05 (16.42, 19.75) | 10.30 (9.23, 11.50) |
| 1996 | 13.80 (12.70, 14.97) | 17.44 (16.01, 18.96) | 9.85 (8.9, 10.98) |
| 1997 | 13.32 (12.28, 14.47) | 16.91 (15.58, 18.47) | 9.42 (8.54, 10.47) |
| 1998 | 12.90 (11.82, 13.91) | 16.46 (15.14, 17.80) | 9.02 (8.09, 9.97) |
| 1999 | 12.30 (11.36, 13.35) | 15.77 (14.56, 17.05) | 8.51 (7.75, 9.41) |
| 2000 | 11.85 (11.03, 12.83) | 15.30 (14.15, 16.65) | 8.10 (7.33, 8.90) |
| 2001 | 11.36 (10.61, 12.39) | 14.74 (13.69, 16.13) | 7.67 (6.96, 8.47) |
| 2002 | 10.99 (10.30, 11.88) | 14.26 (13.29, 15.44) | 7.44 (6.80, 8.23) |
| 2003 | 10.45 (9.75, 11.33) | 13.60 (12.70, 14.70) | 7.04 (6.44, 7.75) |
| 2004 | 10.12 (9.42, 10.85) | 13.20 (12.23, 14.30) | 6.80 (6.29, 7.42) |
| 2005 | 9.51 (8.83, 10.22) | 12.39 (11.50, 13.35) | 6.42 (5.91, 6.97) |
| 2006 | 8.71 (8.09, 9.34) | 11.41 (10.60, 12.28) | 5.81 (5.34, 6.35) |
| 2007 | 8.17 (7.60, 8.78) | 10.70 (9.92, 11.58) | 5.44 (5.00, 6.02) |
| 2008 | 7.90 (7.35, 8.51) | 10.41 (9.60, 11.34) | 5.19 (4.79, 5.71) |
| 2009 | 7.84 (7.33, 8.38) | 10.43 (9.63, 11.24) | 5.04 (4.68, 5.48) |
| 2010 | 7.43 (6.94, 8.01) | 9.88 (9.12, 10.72) | 4.78 (4.39, 5.24) |
| 2011 | 6.88 (6.45, 7.35) | 9.14 (8.47, 9.89) | 4.44 (4.05, 4.82) |
| 2012 | 6.48 (6.02, 6.98) | 8.67 (7.96, 9.51) | 4.12 (3.76, 4.49) |
| 2013 | 6.16 (5.69, 6.64) | 8.29 (7.60, 9.12) | 3.87 (3.53, 4.21) |
| 2014 | 5.86 (5.43, 6.35) | 7.90 (7.25, 8.66) | 3.68 (3.32, 4.04) |
| 2015 | 5.60 (5.12, 6.16) | 7.59 (6.79, 8.51) | 3.47 (3.10, 3.86) |
| 2016 | 5.39 (4.89, 6.01) | 7.33 (6.53, 8.23) | 3.34 (2.96, 3.75) |
| 2017 | 5.15 (4.67, 5.72) | 7.00 (6.17, 7.95) | 3.19 (2.80, 3.64) |
| 2018 | 4.95 (4.42, 5.48) | 6.70 (5.77, 7.66) | 3.08 (2.70, 3.52) |
| 2019 | 4.75 (4.20, 5.35) | 6.43 (5.53, 7.44) | 2.95 (2.51, 3.42) |
| 2020 | 4.31 (3.77, 4.95) | 5.87 (4.95, 6.93) | 2.66 (2.22, 3.10) |
| 2021 | 4.15 (3.62, 4.72) | 5.65 (4.79, 6.63) | 2.56 (2.14, 3.01) |
